# The effect of rapamycin on bovine oocyte maturation success and metaphase telomere length maintenance

**DOI:** 10.18632/aging.103126

**Published:** 2020-04-27

**Authors:** Pawel Kordowitzki, Meriem Hamdi, Aksinya Derevyanko, Dimitrios Rizos, Maria Blasco

**Affiliations:** 1 Institute of Animal Reproduction and Food Research of Polish Academy of Sciences, Olsztyn, Poland; 2 Institute of Veterinary Medicine, Nicolaus Copernicus University, Torun, Poland; 3 Instituto Nacional de Investigación y Tecnología Agraria y Alimentaria (INIA), Department of Animal Reproduction, Madrid, Spain; 4 Telomeres and Telomerase Group, Spanish National Cancer Research Centre (CNIO), Madrid, Spain

**Keywords:** reproductive aging, oocytes, blastocysts, rapamycin, telomere

## Abstract

Maternal aging-associated reduction of oocyte viability is a common feature in mammals, but more research is needed to counteract this process. In women, the first aging phenotype appears with a decline in reproductive function, and the follicle number gradually decreases from menarche to menopause. Cows can be used as a model of early human embryonic development and reproductive aging because both species share a very high degree of similarity during follicle selection, cleavage, and blastocyst formation. Recently, it has been proposed that the main driver of aging is the mammalian target of rapamycin (mTOR) signaling rather than reactive oxygen species. Based on these observations, the study aimed to investigate for the first time the possible role of rapamycin on oocyte maturation, embryonic development, and telomere length in the bovine species, as a target for future strategies for female infertility caused by advanced maternal age. The 1nm rapamycin *in vitro* treatment showed the best results for maturation rates (95.21±4.18%) of oocytes and was considered for further experiments. In conclusion, rapamycin influenced maturation rates of oocytes in a concentration-dependent manner. Our results also suggest a possible link between mTOR, telomere maintenance, and bovine blastocyst formation.

## INTRODUCTION

The female reproductive axis is the first to fail while growing old and is associated with changes in ovarian function in most mammalian species [1, 2]. With regards to that, fertility could be an important issue to explain the fundamental processes of aging [3]. Oocytes may undergo two types of aging. On one hand, an oocyte can be exposed to an aged ovarian microenvironment before being ovulated, known as ‘reproductive or maternal aging’. On the other hand, there is ‘postovulatory aging’, meaning either a prolonged stay in the oviduct or *in vitro* aging before fertilization. However, the molecular mechanisms underlying these aging processes are still poorly understood [4]. Reproductive aging in women and other mammals is associated with a progressive decline of ovarian function characterized by a decrease in the quantity and quality of oocytes with advancing age [1]. In recent decades it has been observed that women in most industrialized societies postpone their first pregnancy [5]. Consequently, the average age of women when they first attempt childbearing has increased [1, 6]. In relation to an increasing number of women delaying childbirth, enormous efforts are being made to diagnose and counteract age-associated infertility. It is well known that fertility in women begins to decline significantly by their mid-30s and pregnancies in advanced age women lead to higher rates of miscarriage and/or an aneuploid offspring [7]. It is generally accepted that chromosomal abnormalities, spindle defects, mitochondrial dysfunction, telomere shortening, and a decrease in the levels of maturation-promoting factor (MPF) are directly implicated in age-related decline of fertility and embryonic development [6, 8–13].

Telomeres are TTAGGG repeats that cap chromosome ends, prevent end-to-end fusions, and shorten with each cell division in most cells until they become critically short and promote cell cycle arrest, apoptosis, and genomic instability [14–17]. Telomeres shorten with age through two mechanisms: replicative senescence in dividing cells and via the effects of reactive oxygen species (ROS) in non-dividing cells such as oocytes [18]. Recently, it has been proposed that the main driver of aging is the target of rapamycin (TOR) signaling rather than ROS. Inhibition of TOR, either pharmacologically with rapamycin or genetically, extends the life span of yeast and *C. elegans* [19–24].

The objective of this project was to study for the first time, the effects of appropriate concentrations of rapamycin on oocyte maturation until the metaphase II (MII) stage, the developmental capacity of bovine oocytes, and their telomere length in order to better understand the link between aging, telomeres, and mTOR signaling. These findings can be applied to bovine and human *in vitro* fertilization (IVF) procedures, it may also aid in overcoming oocyte aging.

## RESULTS

### Influence of different rapamycin concentrations on oocyte maturation

The use of 1nM rapamycin during IVM increased in a most potent, even though not significant, way the maturation rate (95.21±4.18% in metaphase II) of bovine oocytes compared to the control groups. We, therefore, decided to use this concentration for further replicates to investigate if there any effects on early embryonic development (Table 1). Overall a dose-dependent effect of rapamycin could be detected. The concentration 100nM of rapamycin supplementation diminished the maturation rates (82.37±4.88% in metaphase II), and this was of statistical difference when compared to the 1nM Rapamycin supplementation (p=0.0326). Both concentrations, 0.1nM and 10nm, did not differ drastically from the control groups.

**Table 1:** Maturation rates after different rapamycin test concentrations.

**Treatment group**	**COCs in culture total number**	**Maturation rate**	
		**Total number**	**%±SEM**
0.01nM Rapamycin	34	30	87.74±6.74
1nM Rapamycin	38	36	95.21±4.18
10nm Rapamycin	31	28	90.24±1.17
100nm Rapamycin	28	23	82.37±4.88
Control	33	30	90.85±0,83
DMSO/vehicle Control	32	28	87.58±5.01

Maturation rates are shown as total numbers and as percentages (±SEM) of the total number of COCs in culture. Statistical significance (p ±0.05) between the values is indicated by the asterisk.

### Influence of rapamycin on early embryonic development

**Figure 1:**
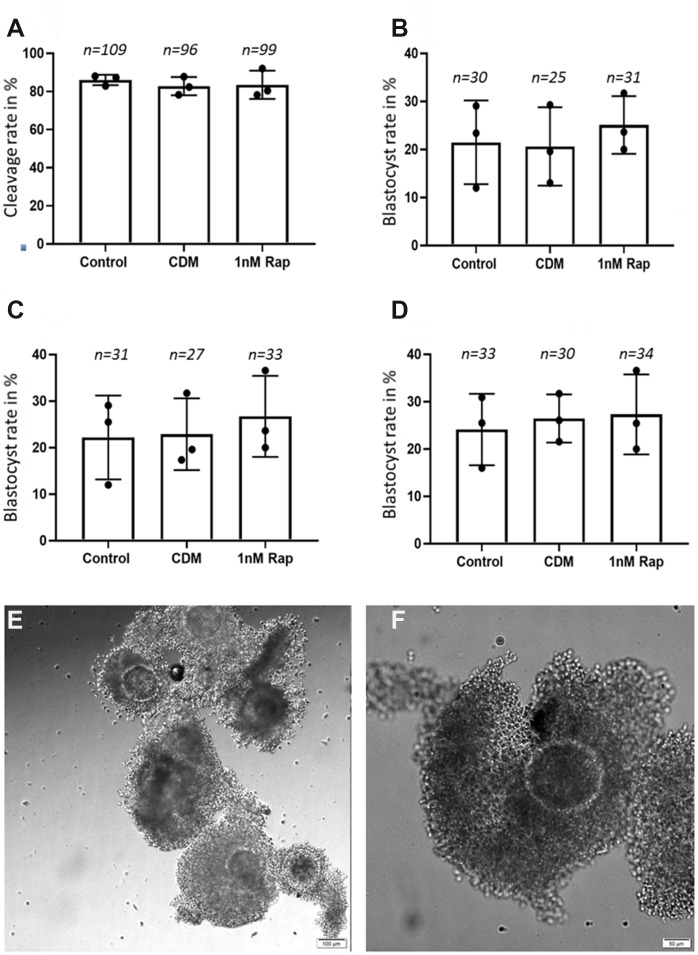
**Influence of 1nM Rapamycin supplementation on early embryonic development.** The results are presented as cleavage rates and blastocyst rates in % (total number of metaphase II oocyte/total number of embryonic stage). (**A**) shows the results for proper cleaved embryos on day 4 in the three experimental groups. (**B**) shows the results for the blastocyst rate on day 7 in the three experimental groups. (**C**) shows the results for the blastocyst rate on day 8 in the three experimental groups. (**D**) shows the results for the blastocyst rate on day 9 in the three experimental groups. A total of 695 good quality COCs (representative COCs shown in (**E**) [scale bar: 100 μm] and (**F**) [scale bar: 50 μm]) were used for the *in vitro* maturation, fertilization, and culture until day 9, and the "n" above each bar represents the number of detected good quality early cleavage stage embryos, (control= control without supplementation, CDM=vehicle control with DMSO supplementation, 1nMRap= 1nM Rapamycin supplementation).

In this set of experiments, we intended to see if 1nM rapamycin supplementation used during IVM, has an effect on early embryonic development until the blastocyst stage. A total of 695 good quality COCs (IETS classes I-III) underwent IVM, following IVF and IVC both of which were performed without rapamycin supplementation. Although there was no statistical significance, the use of 1nM rapamycin during IVM slightly increased the blastocyst yield on day 7; 25.11±1.17% blastocyst after *in vitro* treatment in comparison to 21.5±3.14% and 20.64±2.68% in the control groups (Figure 1).

### Influence of rapamycin on telomere length

**Figure 2:**
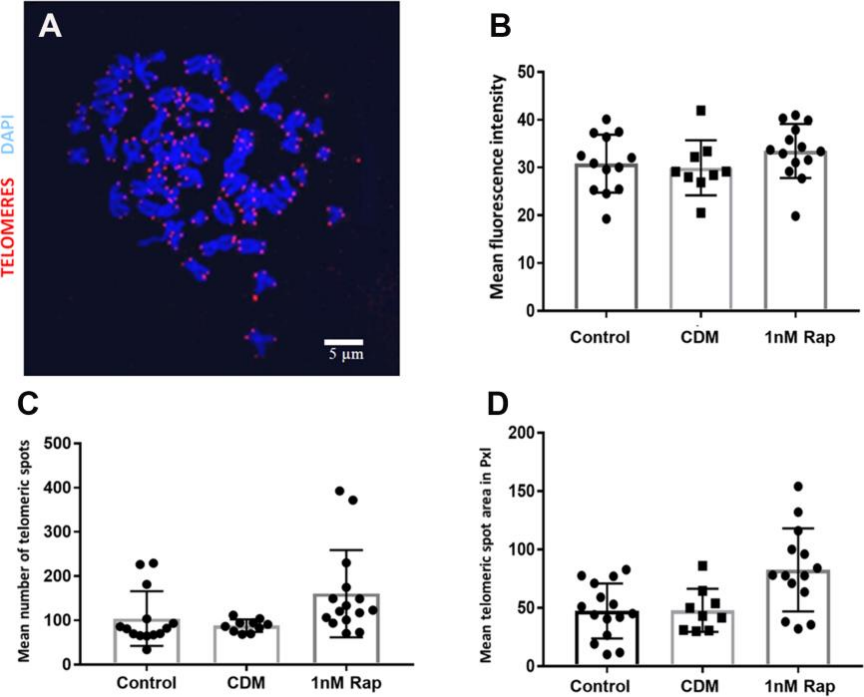
**Telomere visualization and evaluation after Q-FISH in metaphase spreads of bovine oocytes.** Telomeres were detected by Q-FISH on MII oocytes using a probe targeting telomeric repeats (red) and DNA stained with DAPI (blue). (**A**) Oocyte Metaphase II spread, scale bar: 5 μm. (**B**) Mean fluorescence intensity of telomeric spots in all experimental groups. (**C**) shows the results for the mean number of telomeric spots in the three experimental groups. (**D**) shows the results for the mean telomeric spot area expressed in Pixels (pxls) in the three experimental groups. (control= control without supplementation, CDM=vehicle control with DMSO supplementation, 1nMRap= 1nM Rapamycin supplementation).

Due to the possible link of the mTOR pathway to telomeres, we investigated the effect of 1nM rapamycin supplementation on the relative telomere length of metaphase chromosomes in bovine oocytes. Although rapamycin supplementation increased the telomere length of metaphase chromosomes of oocytes, there was no statistical significance when compared to the control groups (Figure 2). Additionally, we examined the number of telomeric spots to estimate a possible link between the rapamycin treatment and telomere aberration. As shown in Figure 2C, 2D the number of telomere spots (129.35 ±54.65) and the telomere spot area expressed in pixels (82.52±34.78) tended to be higher but were not significantly increased compared to the control (104.02±59.57 telomere spots, 35.25±14.62 pxls telomere spot area) and vehicle control groups (87.97±13.48 telomere spots, 47.86±17.42 pxls telomere spot area).

## DISCUSSION

To date, the knowledge of mechanisms on how to delay aging in oocytes is limited, and the pathway of oocyte rescue during aging is poorly described. To investigate for the first time how rapamycin, the mammalian TOR inhibitor and potentially lifespan extender, can influence the quality and telomere length of bovine metaphase II oocytes, the present study focused on the effects of rapamycin during the maturation of oocytes. We chose the bovine species as a model for the human species because of a high degree of similarity in reproductive physiology between both the species. Therefore, cumulus-oocyte-complexes were matured *in vitro* with supplementation of increasing concentrations of rapamycin (0, 0.1, 1, 10, or 100 nM) for 24 h. Since the maturation process is crucial for further biological processes, such as fertilization and early development, and since telomeres are an import hallmark of aging, our study mainly focused on these two topics. Our results demonstrate that maturation rates were highest in the 1nM rapamycin-treated group compared to the control and other treatment groups, whereas in the 100nM rapamycin-treated group fewer oocytes reached the metaphase II stage (Table 1), indicating that rapamycin has a dose-dependent effect. When rapamycin was used at a concentration of 100nM for 12 h, an inhibitory effect on the translation of specific transcripts associated with meiotic progression was observed [25]. In the study where a 1nM rapamycin treatment was chosen for the IVM in porcine oocytes, there were no significant differences observed with regards to the nuclear maturation when compared to the untreated control groups [26]. In that study [26], the 1nM rapamycin supplementation during IVM was used to induce autophagy in porcine COCs. No improvement of oocyte maturation and embryonic development was observed, whereas Song and coworkers [27] described an increase in nuclear and cytoplasmic maturation after the induction of autophagy in porcine COCs. The latest mentioned results are consistent with our findings in bovine oocytes that an *in vitro* treatment with rapamycin tends to improve the rate of metaphase II oocytes. In another study, where a rapamycin treatment was used during porcine oocyte IVM, an increase in the frequency of normal meiotic spindles was observed in the treated group in comparison to untreated control oocytes [26]. Furthermore, in the same study rapamycin led to an increase in the rearrangement of abnormal spindles in aged oocytes. Based on our findings and the data provided by other researchers, we hypothesize that rapamycin positively influences the maturation of oocytes by reaching the second metaphase stage and improves embryonic developmental competence. Since in our experiments rapamycin showed negative effects on maturation rates when concentrations higher than 10nM were used (Table 1), we decided to use only the 1nM concentration group to follow up the early embryonic development until the blastocyst stage to investigate the effects of this exposure during IVM. Although the results are not statistically significant, in our study we could observe a higher blastocyst rate (25.1%) on day 7 in the rapamycin-treated group compared to the two control groups (21.5% and 20.6%). Similar results were obtained in the porcine species, namely, an enhancement of the developmental capacity and quality of blastocysts in the rapamycin-treated group when compared to the controls [26]. Interestingly, in the same study, the relative mRNA abundance of NANOG and SOX2 was higher in blastocysts from the rapamycin-treated oocytes compared to blastocysts from untreated control oocytes. Another study from Lee and coworkers [29] reported that murine blastocysts showed morphological defects after treatment with rapamycin, however, the concentration of rapamycin was 250 times higher than in our study. Furthermore, in the same study, it was demonstrated that rapamycin treatment significantly decreased apoptosis in blastocysts. In a previous study, the same authors reported that autophagy modulators 3-methyladenine and rapamycin could affect the interplay between autophagy and apoptosis in murine embryos [29]. Their results indicate that supplementation of the *in vitro* media with autophagy modulators led to an increase of apoptosis, disrupted mitochondrial morphology, and reduced mitochondrial numbers. These results suggest that rapamycin could play a role as an anti-apoptotic agent and might support the interplay between autophagy and apoptosis during early embryonic development. Based on our results and the provided data from the literature, we hypothesize that rapamycin is a potent compound that seems to increase the *in vitro* oocyte culture outcomes and does not decrease the early embryonic development in bovine species. Furthermore, one should take into account that our study until today is the only one carried out in bovine species.

Another main interest of our study, which has not yet been described before in bovine oocytes, was to examine whether a rapamycin treatment influences telomere length maintenance. The mammalian target of rapamycin (mTOR) is an evolutionarily conserved Ser/Thr protein kinase that controls different cellular events, for instance, cell cycle progression, cell size, transcription, dynamics of the cytoskeleton, and autophagy [30–32]. Previous studies provided strong evidence that the inhibition of the TOR pathway extends lifespan in yeast, worms, and flies [33–35]; another study demonstrated that rapamycin extends lifespan in genetically heterogeneous mice [36, 37]. It has been reported that rapamycin-treated porcine oocytes significantly decreased mTOR protein synthesis and mRNA expression, which indicates that rapamycin is involved in the inhibition of mTOR [26]. In our study, based on the results of the Q-FISH (Figure 1C), we could not detect any statistical differences in telomere length between the rapamycin treatment and the control groups, although in a previous study rapamycin was reported to affect the level of hTERT mRNA in mammals [38]. In our results, we could see a tendency of an increased number of telomeric spots in the rapamycin-treated group, which could give a hint for a possible link to telomeric aberrations such as multi telomeric signals [22, 39]. In the work of Unger and co-workers [40], where the influence of rapamycin on the telomere length in different yeast strains was tested, data provided the hypothesis that the TOR complex 1 (TORC1), which coordinates the response to nutrient starvation and is sensitive to rapamycin [41], plays a crucial role in the control of telomere length. In addition to its role in telomere length maintenance and protection, the TORC1 affects the Ku heterodimer which relocates to sites of double-strand breaks to promote their repair by non-homologous end joining (NHEJ) [42]. Moreover, the Ku heterodimer is important for the telomere length maintenance and is involved in telomere protection and localization as well as in telomerase recruitment. That could be the mechanism by which rapamycin possibly affects telomere length [43–45]. In conclusion, we can summarize that we were the first group to use rapamycin in bovine oocyte *in vitro* maturation, and although the results did not show a significant difference, but there was a tendency that rapamycin is beneficial with regards to embryo development at an appropriate dose. Further investigations are needed to investigate the influence of rapamycin on telomere length.

## MATERIALS AND METHODS

### Media

Unless stated otherwise, all chemicals were purchased from Sigma Aldrich Quıímica (Madrid, Spain).

### Oocyte collection and *in vitro* oocyte maturation (IVM)

Bovine cumulus–oocyte complexes (COCs) were recovered and matured *in vitro* as previously described by Lopera-Vasquez [46]. Briefly, immature COCs were obtained by aspirating follicles (2–8 mm) from the ovaries of heifers collected at the slaughterhouse. Classes I-III COCs (Figure 1E, 1F) were matured for 22 h in groups of approximately 50 COCs per well in four-well dishes (NUNC, Roskilde, Denmark) in 500 μL of *in vitro* maturation medium “IVM” (TCM 199 (M4530) supplemented with 10% (v/v) fetal calf serum (FCS) and 10 ng/mL epidermal growth factor (E4127). In a preliminary experiment, we aimed to detect a possible dose-dependent effect of rapamycin, where four increasing concentrations were assessed: 0.1nM, 1nM, 10nM, and 100nM. The IVM standard media as well as media supplemented with the vehicle “0.0001x of DMSO” were used as controls. The final-used concentration of rapamycin for IVM was determined according to the results of the highest maturation rate obtained after 24h, in comparison to the control groups. In the subsequent experiment, the following groups of IVM were performed: (1) Control: COCs matured in IVM media (2) 1R: COCs matured in IVM media supplemented with 1nM of rapamycin (3) Control DMSO (CD): COCs matured in IVM media supplemented with DMSO as previously. The culture conditions were 38.5°C, 5% CO_2_ air, and maximum humidity.

### *in vitro* fertilization (IVF)

Frozen semen from a single Asturian Valley bull, previously tested for IVF (ASEAVA, Asturias, Spain), was thawed at 37°C in a water bath for 1 min and sperm was selected on a Bovipure gradient (Nidacon Laboratories AB, Gothenburg, Sweden) as previously described by Lopera-Vasquez and coworkers (2017). Sperm concentration was determined and adjusted to a final concentration of 1 × 10^6^ sperm cells/ mL for IVF. Gametes were co-incubated for 18 h in 500 μL of fertilization medium (Tyrode’s medium with 25 mM bicarbonate, 22 mM Na-lactate, 1 mM Na-pyruvate and 6 mg/mL fatty acid-free BSA) supplemented with 10 μg/mL heparin sodium salt (Calbiochem) in groups of 50 COCs per well in four-well dishes at 38.5°C in an atmosphere of 5% CO_2_ at maximum humidity.

### *in vitro* embryo culture

At approximately 20 h after insemination, presumptive zygotes were denuded of cumulus cells by vortexing for 3 min, randomly divided into groups of 25, and cultured in 25 μL droplets of synthetic oviductal fluid (SOF) supplemented with 4.2 mM sodium lactate (L4263), 0.73 mM sodium pyruvate (P4562), 30 μL/mL BME amino acids (B6766), 10 μL/mL MEM non-essential amino acids (M7145), 1 μg/mL phenol red (P0290), and 3 mg/mL bovine serum albumin (BSA; A9647). Droplets were placed under mineral oil at 38.5°C in an atmosphere of 5% CO_2_, 5% O_2,_ and 90% N_2_.

### Assessment of metaphase II oocytes

Oocytes were placed into a 5 μL droplet of 0.1% hyaluronidase solution on a pre-cleaned glass slide at room temperature until the *zona pellucida* had dissolved. Individual oocytes were then rinsed for 5–10 s in a 100 μL droplet of hypotonic solution (1% sodium citrate and 0.02 mg/ml human serum albumin) to induce swelling for clearer visualization of the nucleus. The droplet was allowed to evaporate completely and then two drops of fresh 3:1 methanol/acetic acid were added to fix the nuclei. Slides were air-dried at room temperature and stored at 4°C until performing the Q-FISH protocol.

### Quantitative fluorescence in situ hybridization in metaphase spreads of oocytes

Telomere length analysis was performed on oocyte metaphase spreads, which were hybridized with a PNA-telomeric probe and treated as described in Zijlmans et al. [47]. Images were captured on the confocal ultraspectral microscope Leica TCS-SP5-WLL. Analysis of images was performed using the Definiens Developer XD2 software.

### Statistical analysis

The results for the maturation and developmental rates were analyzed by using the Chi-square test and the Kolmogorov-Smirnoff test. The data on telomere length was analyzed using t-test.
